# SERS Sensing of Bacterial Endotoxin on Gold Nanoparticles

**DOI:** 10.3389/fimmu.2021.758410

**Published:** 2021-10-07

**Authors:** Alessandro Verde, Maria Mangini, Stefano Managò, Chiara Tramontano, Ilaria Rea, Diana Boraschi, Paola Italiani, Anna Chiara De Luca

**Affiliations:** ^1^Institute for Experimental Endocrinology and Oncology, “G. Salvatore” (IEOS), Second Unit, Consiglio Nazionale Delle Ricerche (CNR), Napoli, Italy; ^2^Institute of Biochemistry and Cell Biology (IBBC), Consiglio Nazionale Delle Ricerche (CNR), Napoli, Italy; ^3^Institute of Applied Sciences and Intelligent Systems (ISASI), Unit of Napoli, Consiglio Nazionale Delle Ricerche (CNR), Napoli, Italy; ^4^Department of Biology and Evolution of Marine Organisms, Stazione Zoologica “Anton Dohrn”, Napoli, Italy; ^5^The Shenzhen Institutes of Advanced Technology (SIAT), Chinese Academy of Science (CAS), Shenzhen, China

**Keywords:** SERS, gold nanoparticles, LPS, biosensor, inflammation, innate immunity

## Abstract

Engineered gold nanoparticles (AuNPs) find application in several fields related to human activities (*i.e.*, food and cosmetic industry or water purification) including medicine, where they are employed for diagnosis, drug delivery and cancer therapy. As for any material/reagent for human use, the safety of AuNPs needs accurate evaluation. AuNPs are prone to contamination by bacterial endotoxin (lipopolysaccharide, LPS), a potent elicitor of inflammatory responses in mammals. It is therefore important, when assessing AuNP immunosafety and immune-related effects, to discriminate between inflammatory effects intrinsic to the NPs from those caused by an undeliberate and undetected LPS contamination. Detection of LPS contamination in AuNP preparations poses different problems when using the current LPS detection assays, given the general interference of NPs, similar to other particulate agents, with the assay reagents and endpoints. This leads to time-consuming search for optimal assay conditions for every NP batch, with unpredictable results, and to the use in parallel of different assays, each with its weaknesses and unpredictability. Thus, the development of highly sensitive, quantitative and accurate assays able to detect of LPS on AuNPs is very important, in view of their medical applications. Surface-enhanced Raman spectroscopy (SERS) is a label-free, sensitive, chemical-specific, nondestructive and fast technique that can be used to directly obtain molecular fingerprint information and a quantitative analysis of LPS adsorbed on AuNPs. Within this study, we describe the use of SERS for the label-free identification and quantitative evaluation - down to few attograms - of the LPS adsorbed on the surface of 50 nm AuNPs. We thus propose SERS as an efficient tool to detect LPS on the AuNP surface, and as the basis for the development of a new sensitive and specific LPS-detection sensor based on the use of AuNPs and SERS.

## Introduction

Bacterial endotoxin or lipopolysaccharide (LPS) is the main component of the cell wall of gram-negative bacteria and it is considered a ubiquitous contaminant in the environment, and is resistant to the most common sterilization procedures (1). Human cells are very sensitive to LPS. Upon interaction with membrane receptors (including the Toll-like receptor TLR4, mainly expressed by innate immune cells), LPS triggers the production of inflammatory cytokines such as TNF-α, IL-1β, and chemokines (*e.g.*, CCL2/MCP-1 and CXCL8/IL-8), which all contribute to the inflammatory process (2). One of the most severe effects of LPS in human beings is the septic shock syndrome, a life-threatening disease that accounts for 20% of all global deaths each year (3). For this reason, before being marketed and used in biomedical fields, every drug or biomedical device must undergo accurate testing to determine LPS contamination. The maximum tolerated LPS level in drugs or surgical instruments for human use is 0.5 EU/kg, whilst, if the product comes in contact with the cerebrospinal fluid, the limit is 0.02 EU/kg (4).

In the last years, engineered nanoparticles (NPs) have gained increasing attention in the biomedical field. In particular, gold NPs (AuNPs), owing to their chemical and optical properties, are used in a wide range of different applications in medicine (*e.g.*, drug delivery, diagnostic and imaging procedures) (5–9). As for every biomedical device, also AuNPs must be carefully tested for their immunosafety (inability to trigger immune/inflammatory reactions) and for the presence of LPS contamination. As for many other nanomaterials, AuNPs are very prone to LPS contamination: on one side, because of its amphipathic nature LPS can easily bind NPs with either a cationic or anionic surface; on the other side, LPS binding onto NP surface is facilitated by the high NP surface to volume ratio that increases NP reactivity towards LPS (10). The detection of LPS contamination on AuNPs is crucial for the assessment of particle toxicity and biological properties avoiding misinterpretation of the AuNP impact on human health.

The LPS contamination in pharmaceutical preparations, including AuNPs, is currently detected using regulatory-approved assays: the Limulus amoebocyte lysate (LAL) assay, the monocyte activation test (MAT) and the rabbit pyrogen test (RPT). The application of these assays to the detection of LPS adsorbed on the AuNP surface is not easy, mainly because NPs can interfere with these assays, leading to unreliable results (11–13). Among these methods, the LAL test is the most specific towards LPS. It is based on the capacity of LPS to activate Factor C, an enzyme present in the amoebocytes of the marine arthropod *Limulus polyphemus*. The LAL assay is commercially available in different formats (turbidimetric, gel-clot or chromogenic LAL assays, and most recently the assays based on recombinant factor C). Although LAL assays have a good sensitivity (0.005 EU/mL), NPs can interfere with the assay components or final readout. For this reason, the choice of the most suitable LAL assay is empirical and it can vary case by case (11), or it may be necessary to run different assays in parallel to validate the results (13). Except for the recombinant Factor C assays, all the traditional LAL assays based on the amoebocytes of *L. polyphemus* will soon be abandoned by the regulatory authorities worldwide because of the harm caused to the animals. The RPT and MAT assays measure the response of rabbits *in vivo* or human monocytes *in vitro*, respectively, to inflammatory agents. Their main disadvantage is that they are pyrogen assays, not specific for LPS but sensitive to every inflammatory or pyrogenic agent. Aside from the fact that RPT and MAT are not LPS-specific, particular attention has to be paid to the NP concentration used in these assays to avoid non-specific toxicity for animals or cells. Moreover, these tests are not suitable for NPs carrying cytotoxic drugs. Eventually, as in the case of the LAL assays, the RPT assay will soon be abandoned because of the use of animals. For all these reasons, it is clear that none of the current LPS detection assays is fully suitable for assessing the LPS presence on AuNPs, and that new methods are needed for the reliable and quantitative detection of LPS.

Surface enhanced Raman spectroscopy (SERS) represents an excellent tool for molecular detection, because it can amplify the Raman signals of a given molecule adsorbed onto metallic nanosurfaces, as gold or silver NPs (14). Extraordinary sensing properties of SERS can be achieved by exploiting the metallic NPs’ optical amplification mediated by localized surface-plasmon resonances. When an incident laser beam interacts with metallic nanostructures, plasma oscillations of metallic NPs enhance the Raman signals of molecules adsorbed or close to the metallic surface up to 6-7 orders of magnitude (15). Therefore, due to the Raman fingerprint, SERS-based sensing allows to identify and quantify molecules with excellent sensitivity and reproducibility in different environments, thereby enabling the use of the SERS technique for numerous biomedical and biosensing applications (16–20). Indirect SERS sensing, based on the combined use of a SERS tag and a selective aptamer-based binding, has been demonstrated for quantitative detection of endotoxins in a solution (21). Additional advantages of the SERS technique are that it does not require labelling or other specific treatments for sample preparation, measurements can be performed on few μL of desired NP concentration, and the technique is non-destructive, allowing for performing multiple measurements on the same sample. Based on such considerations, the SERS is a good candidate for label-free detection of even minute amounts of LPS directly on metallic NPs.

In this study, we demonstrate the use of the SERS approach for the quantitative detection of LPS contamination in AuNPs. We describe the use of SERS for the direct chemical identification and quantitative evaluation of LPS adsorbed on the surface of 50 nm AuNPs. DLS was used to estimate the number of LPS molecules adsorbed on the surface of AuNPs previously exposed to saturating concentrations of LPS. LPS concentration-dependent SERS signals were collected in order to quantify LPS molecules on AuNPs at low concentration and define the limit of detection (LOD).

## Materials and Methods

### Gold Nanoparticles

LPS-free nanoparticles consisting of 50 nm gold nanospheres (AuNPs) were purchased from Applied Nanoparticles S.L. (Barcelona, Spain). AuNPs are diluted in a 2.2 mM sodium citrate solution at a concentration of 1 mg/mL (corresponding to about 7*10^11^ NPs/mL) and stored in sterile endotoxin-free plastic tubes. According to the data sheet, the endotoxin contamination level of these AuNPs was determined to be less than 0.25 EU/mL (roughly corresponding to 25 pg/mL) by the chromogenic LAL assay.

### LPS Adhesion to AuNPs

Gel filtration chromatography-purified LPS from *Escherichia coli* O55:B5 (catalogue number L6529) and phenol extraction-purified LPS from *Klebsiella pneumoniae* (catalogue number L4268-10MG) were both obtained by Sigma-Aldrich (Merck KGaA, St. Louis, MO, USA).

Two μL of 50 nm AuNPs (at 1 mg/mL) were incubated with 1 μL of LPS (from *E. coli* or *K. pneumoniae*) at different concentrations (from 0.1 to 5000 μg/mL) in a total volume of 50 μL of 2.2 mM sodium citrate solution for 20 min at 37°C, leading to a stable LPS corona formation (22). In order to remove the non-adsorbed LPS excess, the products were washed twice in sodium citrate solution by centrifugation (22,000 *x g*, 10 min, RT), and pellets were resuspended in sodium citrate solution or cell culture medium, depending on the expected use.

### Transmission Electron Microscopy (TEM)

For morphological analysis, an aliquot of bare or LPS-coated AuNPs was drop-casted on a carbon-coated copper grid and allowed to dry at room temperature (RT). TEM images were acquired with an accelerating voltage of 120 kV using a FEI Tecnai 12 Bio Twin Spirit TEM (FEI Company, Hillsboro, OR, USA). Images of bare and LPS-coated AuNPs were analyzed and measured for size distribution using the ImageJ software (Wayne Rasband, NIH, Bethesda, Maryland, USA). Size distribution data were averaged, in order to obtain a mean size both for bare and LPS-coated AuNPs, and their standard deviations (SDs) were calculated.

### Dynamic Light Scattering (DLS)

Hydrodynamic diameter and ζ-potential of bare and LPS-AuNPs were measured with a ZetaSizer Nano ZS (Malvern Instruments, Malvern, Worcestershire, UK). The instrument operates with a 633 nm He-Ne laser wavelength and a fixed scattering angle of 173°. For measurements, bare or LPS-AuNPs (20 μL at a concentration of 100 μg/mL) were suspended in 1 mL of distilled water. Data obtained from hydrodynamic diameter and ζ-potential measurements were averaged in order to obtain a mean size and SD both for bare and LPS-coated AuNPs.

The amount of LPS in the corona around AuNPs was estimated as previously described (22). Briefly, the volumes of bare and LPS-coated AuNPs were estimated considering as radius the measured hydrodynamic diameters (sphere model). LPS corona volumes were then obtained by subtracting bare AuNP volumes from concentration-dependent LPS-coated AuNP volumes. Being known the LPS density *ρ_LPS_* (1.44 g/cm^3^) and the average LPS molecular weight *M_LPS_* (20 kDa), the LPS corona masses were estimated by:


(1) $$m_{LPS\;corona}=V_{LPS\;corona}*\rho_{LPS}\;$$

where:


(2) $$V_{LPS\;corona}=V_{NP+LPS}-V_{NP}=\frac{4}{3}\pi \left(R_{NP+LPS}^{3}-R_{NP}^{3}\right)$$

*V_NP_*_+_*_LPS_* being the volume of LPS-coated AuNPs and *V_NP_* the volume of bare AuNPs. *R_NP_*_+_*_LPS_* and *R_NP_* are hydrodynamic radii of LPS-coated AuNPs and bare AuNPs, respectively.

The number of LPS molecules was then estimated as follows:


(3) $$LPS\;molecules=m_{LPS\;corona}/M_{LPS}$$

### Raman and Surface-Enhanced Raman Spectroscopy and Spectra Analysis

Raman and SERS spectra were acquired with an inverted confocal Raman microscope (XploRA INV, HORIBA Jobin Yvon S.A.S., Villeneuve d’Ascq, France) equipped with a 785 nm wavelength diode laser (23). A 60x/1.2 NA water immersion objective (Nikon) was used to focus the laser light onto the sample and collect the Raman signal. The back scattered light from the sample was spectrally filtered by a notch filter and then directed towards a spectrometer equipped with a holographic grating (600 lines/mm). Finally, Raman signal was detected by a thermoelectrically cooled CCD detector. The pinhole (500 μm) and spectrometer entrance slit (200 μm) were selected to ensure an in-plane spatial resolution (x-y plane) of about 400 nm and 5 μm along z axis.

For Raman experiments, the LPS powder was deposited on a CaF_2_ coverslip (Crystran Ltd., Poole, UK), and then the laser was focused onto the sample. Raman spectra were acquired with 8.8 mW laser power at the sample and 30 s integration times per spectra.

For SERS experiments, LPS-coated AuNPs were drop-casted onto a CaF_2_ coverslip (Crystran Ltd.) and let dry at RT. SERS spectra were acquired with 0.9 mW laser power and 1 s integration time. The Raman and SERS spectra of the LPS were averaged and background corrected by creating a baseline with the 2nd Derivative method and then subtracting it with the Peak Analyser function available in OriginLab (OriginLab Corporation, Northampton, MA, USA).

### Enhancement Factor (EF)

To provide an estimate of the signal amplification experienced by each molecule on the nanostructure, we calculated the enhancement factor (EF) (15). A Byphenil-4-Thiol (BPT) solution in ethanol at a concentration of 1 mM was used for EF estimations. A drop (2 μL) of 50 nm AuNPs (concentration at 1 mg/mL) was drop-casted on a CaF_2_ slide and let to dry. By incubating the SERS slide in the BPT solution for 24 h, thanks to the thiol groups showing a strong affinity with the surface, a uniform self-assembled monolayer of BPT molecules on gold surface is obtained (24).

The EF was calculated as:


(4) $$EF=\frac{I_{SERS}}{I_{Raman}}\frac{N_{Raman}}{N_{SERS}}$$

where *I_SERS_* and *I_Raman_* are the intensities of the SERS and Raman signals normalized to the different laser powers and integration times, *N_Raman_* and *N_SERS_* are the number of probed molecules.

*N_Raman_* can be defined as follows:


(5) $$N_{Raman}=\frac{A_{Laser}\;h\;\rho_{BPT}\;N_{A}}{M}$$

where *A_Laser_* indicates the area of the laser spot size (waist of ∼ 800 nm) and *h* is the Rayleigh length (∼ 10 µm). BPT density (1.1 g/cm^3^) is indicated with *ρ_BPT_* while *N_A_* and *M* are Avogadro constant (6.022*10^23^ mol^-1^) and BPT molecular weight (186.3 g/mol), respectively.

On the other hand, *N_SERS_* can be defined as:


(6) $$N_{SERS}=\pi R^{2}A_{Laser}\;\sigma_{NPs}\sigma_{BPT}$$

where *πR*^2^ is the area occupied by a 50 nm AuNP when lying on a surface (*R is the radius*), *σ_NPs_* is the 50 nm AuNPs packing density, *i.e.*, number of AuNPs per area unit (4*10^-5^/nm^2^), in this case the area analysed by Atomic Force Microscopy (AFM)l (**Supplementary Figure 1**) and *σ_BPT_* is BPT packing density (considered to be 4 molecules/nm^2^) (25).

### Limit of Detection (LOD)

Signal to noise ratio (SNR), at each LPS concentration, is a measure related to the quality of the signal and can be defined as:


(7) $$SNR=\frac{I_{Signal}}{I_{Noise}};1\leq SNR$$

where *I_Signal_* is the average intensity of the SERS peak at 1610 cm^-1^ an *I_Noise_* is the noise intensity, evaluated as the SD in the spectral region between 1700 and 1800 cm^-1^ (26). In order to estimate limit of detection (LOD) of our system, which is the concentration at which SNR becomes equal to unity, we focused our attention on the linear region of SNR *vs.* LPS concentration trend and performed a linear fit of the experimental data.

### Human Monocyte Isolation and Macrophage Differentiation

Human peripheral blood mononuclear cells (PBMCs) were isolated from buffy coats of healthy donors by Ficoll-Paque gradient density separation (GE Healthcare, Bio-Sciences AB, Uppsala, Sweden). CD14^+^ monocytes were separated from PBMCs using magnetic microbeads conjugated with an anti-CD14 antibody (Miltenyi Biotec, Bergisch Gladbach, Germany) according to the manufacturer’s instructions. Cell viability was assessed by trypan blue dye exclusion and found to be >95%. Monocytes were plated at 350,000 cells/well in a 24-well plate and cultured in RPMI-1640 + Glutamax-I medium (GIBCO by Life Technologies, Paisley, UK) supplemented with 5% heat-inactivated human AB serum (Sigma-Aldrich) and 50 μg/mL gentamicin sulfate (GIBCO). Cells were cultured in a humidified atmosphere of 5% CO_2_ and at 37°C. Monocytes were differentiated into macrophages in the presence of 50 ng/mL macrophage colony-stimulating factor (R&D Systems, Minneapolis, MN, USA) for 7 days, refreshing the medium every other day. Differentiation into macrophages was confirmed morphologically and by decreased expression of CD14 and increased expression of CD206 compared to monocytes. Macrophages were treated with LPS, bare AuNPs or LPS-AuNPs for 24 h. After treatment, cells were fixed for 15 min at RT in 4% paraformaldehyde (v/v) for morphological analysis, while culture supernatants were collected and stored at -80°C until testing for cytokine production.

### Analysis of Cell Morphology

After fixation, macrophages were permeabilized in 0.02% saponin, 0.5% BSA, and 50 mM ammonium chloride in PBS (blocking solution). Actin cytoskeleton was stained using 33 nM Alexa488-labelled phalloidin (Life Technologies), while nuclei were stained with 2 μg/mL Hoechst 33258 (Sigma Aldrich). Cells were then washed 3x with PBS, and the coverslips were mounted and examined by confocal microscopy (LSM 510; Zeiss, Oberkochen, Germany). Untreated and LPS-activated cells were blind-counted in 30 random fields for each coverslip. Cells were defined as “not activated” when their morphology was regular and without filopodia, and “activated” when filopodia were present on the cell surface.

### Cytokine Analysis

The production and release of the inflammatory cytokine TNF-α, the anti-inflammatory factors IL-1Ra and IL-10, and the chemokine CCL2 was assessed by ELISA (R&D Systems) in the culture supernatants, following the manufacturer’s instructions. Absorbance of assay wavelength was measured at 450 nm using a Cytation 3 imaging reader (BioTek, Winooski, VT, USA). ELISA data were analyzed by GraphPad Prism 9 (GraphPad Inc., La Jolla, CA, USA), and are presented as percentage, considering LPS 10 ng/mL as 100%. Results are reported as mean ± SD of values from different donors/experiments. Statistical significance of differences is indicated by *p* values, calculated using a paired Student’s two-tail *t* test.

## Results and Discussion

### AuNP Morphological Characterization

**Figure 1:**
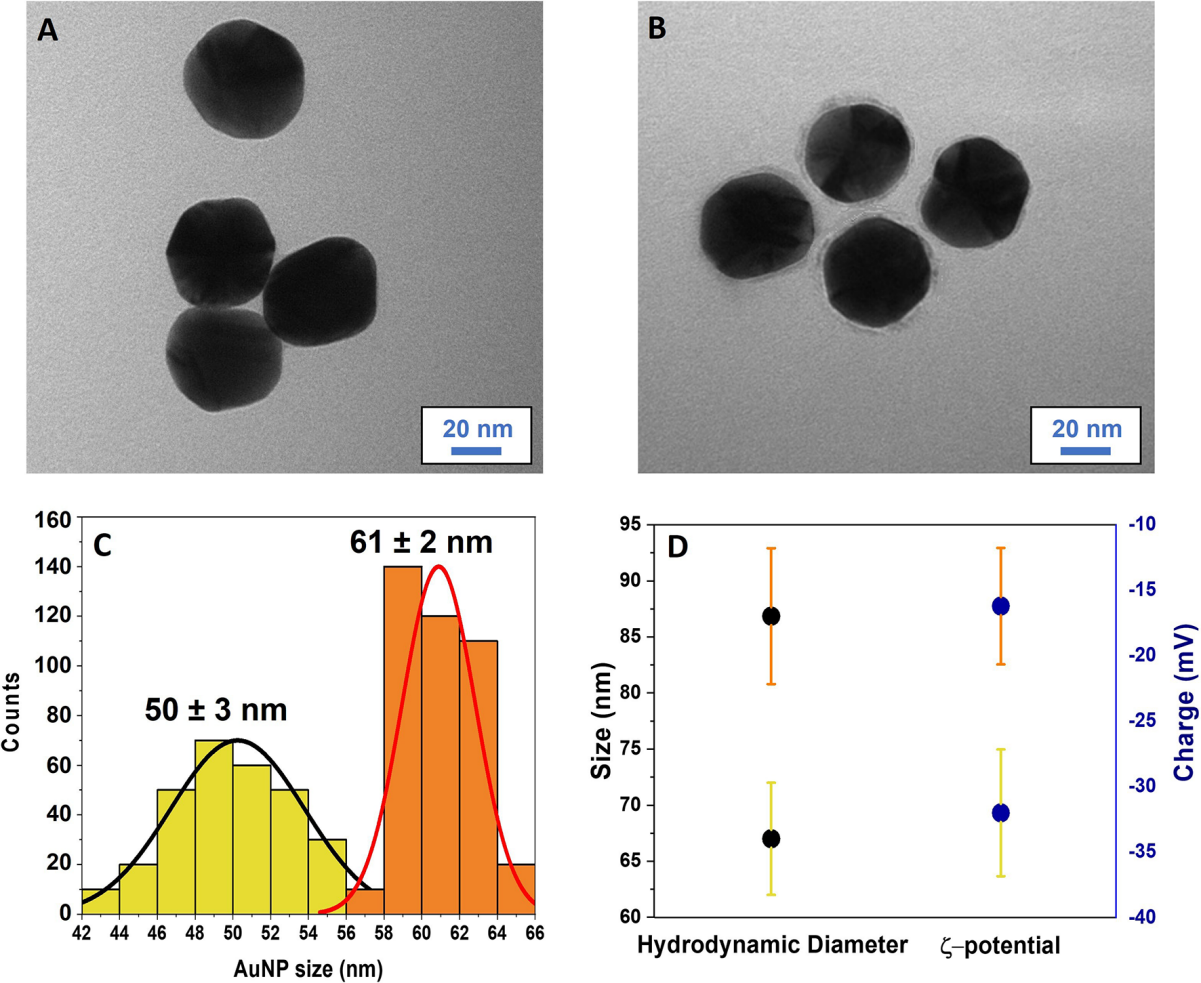
Morphology analysis of the AuNPs and estimation of the LPS corona. TEM images of bare AuNPs **(A)** and LPS-coated AuNPs **(B)**. The LPS corona can be observed as a gray halo around AuNPs. Scale bar: 20 nm. **(C)** Size histograms and Gaussian distribution (black and red lines) related to bare (yellow) and LPS-coated AuNPs (orange). Counts were obtained by measuring the AuNP size in the TEM images. **(D)** Comparison between hydrodynamic diameters and ζ-potentials of bare (yellow SDs) and LPS-coated AuNPs (orange SDs). Results are reported as mean ± SD of data from 5 independent experiments.

Both bare and AuNPs incubated with 5 μL of LPS at a concentration of 1 mg/mL (LPS-AuNPs, see *Materials and Methods* section) were morphologically characterized by TEM, and representative images are reported in **Figure 1**. Bare AuNPs show a diameter of 50 ± 3 nm (**Figure 1A**), confirming the nominal diameter at synthesis. On the other hand, LPS-AuNPs have a diameter of 61 ± 2 nm (**Figure 1B**), corresponding to an increment of 22% compared with bare AuNPs (**Figure 1C**). The increment in the LPS-AuNP diameter is indicative of the LPS adhesion to the NP surface. This finding is also confirmed by TEM images of the LPS-AuNPs, in which it is possible to observe a bright gray halo around NPs, suggestive of an LPS corona with a thickness of ∼ 5 nm. Although TEM provides the morphological characteristics of AuNPs, their quantitative analysis for statistical evaluation is extremely difficult and operator dependent, thus highly variable. For these reasons, we have provided an additional evaluation with DLS.

### DLS Measurements and Estimation of the LPS Corona

**Figure 2:**
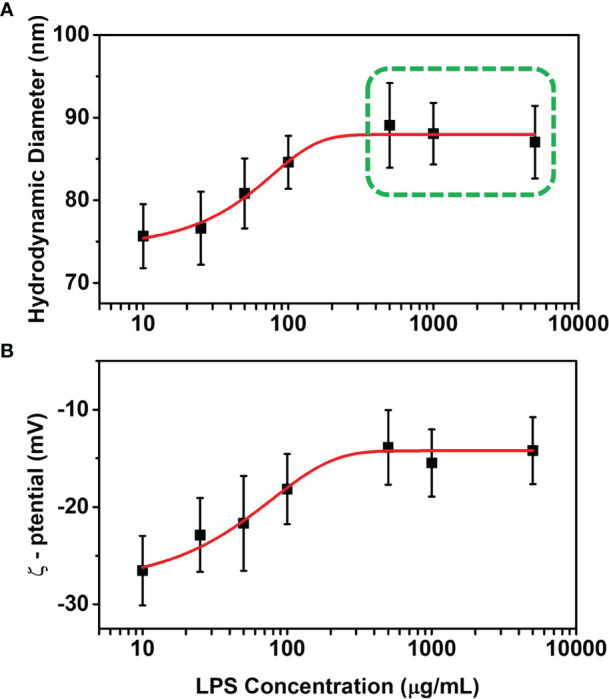
DLS analysis and estimation of the LPS corona. Hydrodynamic diameter **(A)** and ζ-potential **(B)** of AuNPs incubated with different LPS concentrations. The hydrodynamic diameter of bare AuNPs is 67 ± 5 nm, and their ζ-potential is -32 ± 5 mV (see ). In panel A, hydrodynamic diameter values used for LPS corona estimation on 50 nm AuNPs in saturation condition are highlighted with a green dashed square. Results are reported as mean ± SD of data from 4 independent experiments.

**Figure 3:**
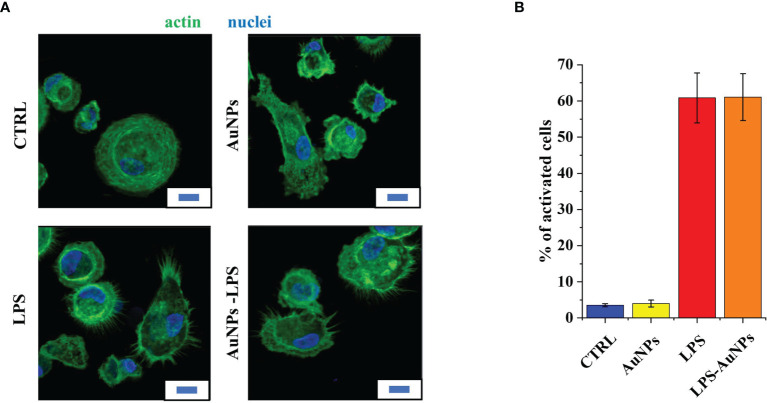
LPS-coated AuNPs induce morphological changes in macrophages. **(A)** Confocal microscopy images showing the morphology of human monocyte-derived macrophages either untreated (CTRL) or exposed to 200 ng/mL free LPS, 1 μg/mL bare AuNPs (AuNPs) and 1 μg/mL LPS-coated AuNPs that contain 200 ng/mL LPS (LPS-AuNPs). The filamentous actin is stained in green, cell nuclei in blue. Scale bar: 10 μm. **(B)** Quantitative evaluation of cells with an activated phenotype for each treatment. Total cell counts for the 3 independent experiments: 2890 cells in the CTRL sample; 2744 cells in the free LPS sample; 2777 cells in the AUNP sample; 2779 cells in the LPS-AuNP sample. Mean ± SD of data from 3 independent experiments.

DLS measurements were performed in order to characterize the hydrodynamic diameter and ζ-potential changes of AuNPs when incubated with different LPS concentrations. First, we performed the DLS measurements by incubating 2 μL AuNPs (1 mg/mL) with 5 μL LPS (1 mg/mL), in order to ensure the saturation of the NP surface with LPS. The hydrodynamic diameter and ζ-potential of the LPS-coated AuNPs were then compared to those of bare AuNPs (**Figure 1D**). The hydrodynamic diameters of bare and LPS-coated AuNPs were 67 ± 5 nm and 87 ± 6 nm, respectively, while their ζ-potentials were -32 ± 5 mV and -16 ± 4 mV, confirming the LPS corona formation around AuNPs. We then measured the hydrodynamic diameters and ζ-potentials of AuNPs incubated with different concentrations of LPS. The trends of the hydrodynamic diameter and ζ-potential as a function of the LPS concentration are reported in **Figures 2A, B**, respectively. A dose-response function was used to fit both hydrodynamic diameters and ζ-potentials data (27). The hydrodynamic diameter shows a linear increase in the LPS concentration range (10 -100 μg/mL) and reaches a saturation for concentration higher than (500 μg/mL). We suppose that the AuNPs size in the linear range increases linearly with the number of molecules of LPS covering the surface of the AuNPs, allowing for the formation of a surface layer that becomes uniform in the saturation region. When the curve reaches a plateau value (500 -5000 μg/mL), the LPS covers the whole NP surface and a uniform LPS layer coating the AuNPs can be hypothesized (saturation of the AuNP surface). As far ζ-potentials are concerned, **Figure 3B** additionally confirms the adhesion of LPS on the NP surface and a saturation behavior for concentration up to 500 μg/mL. Similar to size, when the LPS layer covering the AuNPs becomes uniform, no further variations of the ζ-potential are detectable. The hydrodynamic diameters measured by DLS in saturation conditions (500 - 5000 μg/mL) were used for estimating the number of LPS molecules composing the uniform corona (22). Estimations of LPS corona volumes and number of LPS molecules in it are reported in the **Supplementary Tables 1**, **2**. The number of LPS molecules composing the LPS corona around a single AuNP in saturation conditions was estimated to be 9000 ± 2000. Finally, the total amount of LPS that could bind to 1 μg of 50 nm AuNPs was estimated by multiplying the LPS corona mass for the number of AuNPs used in the experiments, corresponding to ∼10^9^ NPs. Thus, the total amount of LPS adhering to 1 μg of AuNPs at saturation was estimated to be 200 ± 50 ng.

### Biological Effects of LPS-AuNPs on Primary Human Macrophages

In order to verify if the DLS estimation of the amount of LPS bound to AuNPs was reliable, we measured the LPS activity in biological assays, by assessing the activation of innate immune cells. In particular, we used human monocyte-derived macrophages, which are particularly sensitive to LPS (compared to mouse cells or transformed cell lines). First, we examined by confocal microscopy the effect of bare and LPS-coated AuNPs on the macrophage actin cytoskeleton morphology (appearance of lamellipodia and filopodia) (28). **Figure 3** shows the morphology of control untreated macrophages and of macrophages exposed to 200 ng/mL LPS, 1 μg/mL bare AuNPs and 1 μg/mL LPS-coated AuNPs (the amount of LPS of AuNPs calculated as 200 ng, as shown above). Bare AuNPs did not cause morphological changes in macrophages *per se* (**Figure 3**). On the other hand, LPS-coated AuNPs caused the production of filopodia in the same measure as the corresponding concentrations of free LPS (**Figure 3**).

**Figure 4:**
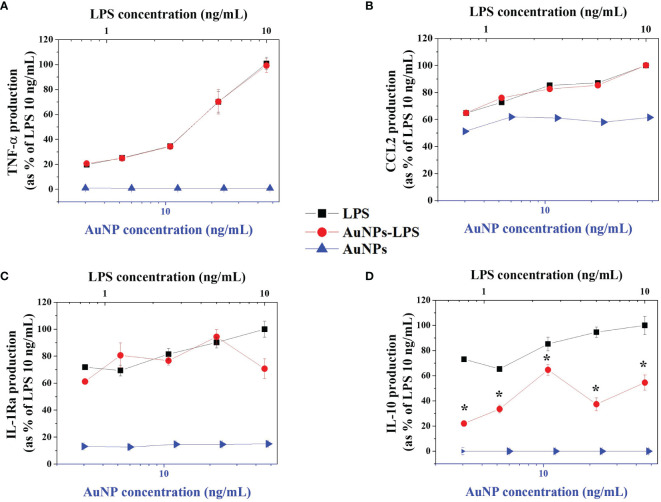
Macrophage activation by LPS-coated AuNPs. Human monocyte-derived macrophages were treated for 24 h with free LPS (black squares), bare (AuNPs; blue triangles) and LPS-coated AuNPs (AuNPs-LPS; red dots). The production of TNF-α **(A)**, CCL2 **(B)**, IL-1Ra **(C)** and IL-10 **(D)** was measured in the culture supernatants by ELISA and normalized as percentage of the maximum value (cytokine levels produced in response to 10 ng/mL LPS). Cytokine concentration ranges were: 0.08-9.40 ng/mL for TNF-α; 0.18-0.38 ng/mL for CCL2; 10.23-78.0 ng/mL for IL-1Ra; 0.28-1.27 ng/mL for IL-10. Results are reported as mean ± SD of data from 3 independent experiments performed on cells from 3 individual donors. *p < 0.01 LPS *vs.* LPS-AuNPs (Student’s *t* tests).

An extensive comparison of free *vs.* NP-bound LPS was performed by examining the inflammatory macrophage activation in terms of production of inflammation-related cytokines in response to increasing concentrations of LPS either free or bound to AuNPs. As shown in **Figure 4**, bare AuNPs did not affect the basal production of the inflammatory factors TNF-α (**Figure 4A**) and CCL2 (**Figure 4B**). Conversely, LPS-coated AuNPs induced the production of TNF-α and of CCL2 with an activation profile that fully overlaps with that induced by free LPS. These results indicate that the DLS estimation of the quantity of LPS bound to NPs was realistic. The fact that LPS attached on the surface of AuNPs can induce human macrophage activation with the same concentration-dependent profile as free LPS supports the hypothesis that association to AuNPs does not change the LPS structure in a way that hampers its binding to macrophage receptors and consequent inflammatory activation.

Since LPS-induced inflammatory activation is self-regulating, with the concomitant induction of anti-inflammatory mechanisms (29), we also tested the production of two important anti-inflammatory cytokines, IL-10 and IL-1Ra. As for the other cytokines, bare AuNPs have no effect on the basal levels of IL-1Ra (**Figure 4C**) and IL-10 (**Figure 4D**), while LPS is active in inducing the production of both anti-inflammatory factors. Notably, while exposure to LPS-AuNPs induced a production of IL-1Ra superimposable to that induced by free LPS, LPS-AuNPs were significantly less active than LPS in inducing the production of the other anti-inflammatory cytokine IL-10. We may hypothesize that, while not affecting the activation of plasma membrane TLR4 and its MyD88-dependent activation pathway, the LPS-coated particles may interfere with the intracellular trafficking of TLR4, in particular with the step of LPS-induced endocytosis of TLR4 and the endocytosis-dependent stimulation of the TRIF pathway. Indeed, the LPS-induced production of TNF-α and IL-1Ra is dependent on the MyD88-initiated pathway, whereas IL-10 production is consequent to the production of type I IFN, which is induced by LPS through the TRIF pathway (30). Recently, it has been reported that the E3 ubiquitine ligase TRIM29 is a negative regulator of type I IFN production (31), suggesting the possibility that LPS-AuNPs may inhibit the production of IL-10 through the enhancement of the expression/activity of TRIM29 and consequent decrease of type I IFN production.

It is important to consider that the biological activity of LPS is different depending on its concentration. As also shown in the results reported in **Figure 4**, the induction of potent inflammatory factors requires higher LPS concentrations, in support of the hypothesis that inflammation starts only as reaction to a substantial threat, while the regulation of more homeostatic defensive factors occurs in response to much lower LPS amounts. An example is the inflammatory cytokine IL-1β, whose gene is upregulated in response to as little as 10 pg/mL LPS but that needs much higher LPS concentrations (2-3 ng/mL) for the maturation and secretion of the active inflammatory protein (32). In **Figure 4**, it is evident that high LPS concentrations are necessary for inducing the production of the potent inflammatory factor TNF-α, while at the lowest concentration used (0.65 ng/mL) induction of TNFα is minimal but production of the anti-inflammatory factors IL-1Ra and IL-10 is already at plateau. This implies that a very low number of LPS molecules on NPs can still trigger significant activation of human innate immune cells. An additional issue is that different LPS, coming from different bacteria, have a different inflammation-inducing capacity. LPS detection bioassays provide results in terms of “Endotoxin Units” (EU), based on biological activity (activation of Factor C in the case of LAL assays), while the correspondence between EU and ng is approximate: the conventional correspondence of 1 EU equal to 100 pg holds almost true for the LPS extracted from the *E. coli* serotype O111:B4 but can be 10-100x higher or lower for other LPS. These considerations underline the need of developing an LPS detection assay that could allow us to measure even very low amounts of LPS adsorbed on NP surfaces, to assess the features of their interaction (in order to evaluate possible structural changes) and, very importantly, to distinguish between different LPS. The development of a SERS assay with such characteristics is described below.

### Raman and SERS Characterization of LPS Molecules on AuNPs

AuNPs are able to amplify the Raman signals by localized surface plasmon resonance, and these unique optoelectronic properties were used as detection probe for LPS.

To characterize the sensing performances of the AuNPs, we preliminary calculated the enhancement factor (EF) using the BPT as model analyte. We recorded 100 spectra from different locations of the substrate to create a statistically significant relevant data distribution. According to the eq. 4, our 50 nm AuNPs show an excellent enhancement capacity, with an EF of about 10^7^ and a good signal uniformity (see **Supplementary Figure 2**).

**Figure 5:**
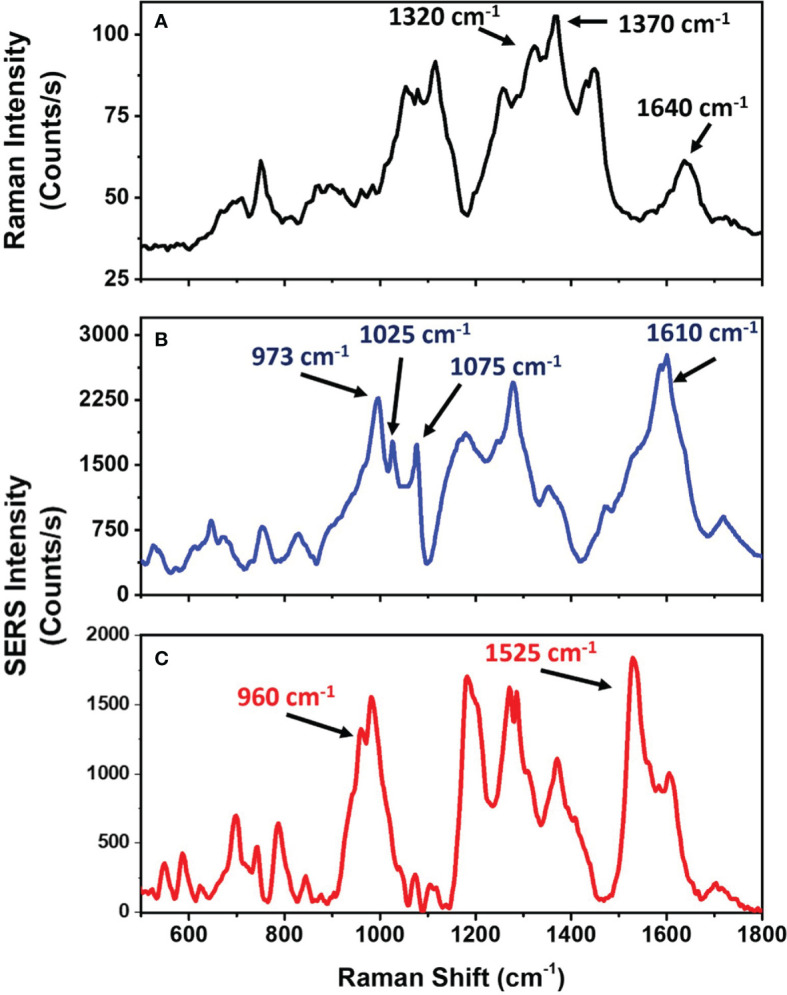
Raman and SERS spectra of LPS from *E*. *coli* and *K*. *pneumoniae*. **(A)** Raman spectrum of LPS powder from *E. coli*. **(B)** SERS spectrum of 50 nm AuNPs incubated with 1 mg/mL of LPS from *E*. *coli*. **(C)** SERS spectrum of 50 nm AuNPs incubated with 1 mg/mL of LPS from *K*. *pneumoniae*.

Because the intensity enhancement is one of the major interests in direct SERS, we investigated the amplification of the LPS Raman profile using SERS technique. **Figure 5** shows a comparison between Raman and SERS spectra of LPS from *E. coli* O55:B5 at a concentration of 1 mg/mL (**Figures 5A, B**). A clear SERS fingerprint of the bacterial endotoxin, similar to its Raman counterpart and in agreement with what found by other groups, is observed. The SERS bands show a shift in frequency compared to Raman ones, which could be ascribed to the specific group absorbed on AuNPs and its orientation respect to the Au surface (33–35). A Raman line broadening during the SERS process is additionally observed, depending from both intermolecular interactions or slightly varying interaction on the AuNPs (35, 36). The LPS molecule is structurally composed by three different parts: a lipid domain called Lipid A, an oligosaccharide core and an extracellular polysaccharide domain called O-antigen (37). Typical bands associated with the O-antigen and core components of the bacterial endotoxin are at 1300–1320 cm^-1^ and 1350–1370 cm^-1^, which can be associated to deformation vibration of C-H bond mode (38). The strongest SERS bands are placed at 973 cm^-1^ and 1610 cm^-1^ and can be related to the core or Lipid A units of the molecule. The first band can be ascribed to the CH bending/C-O-C stretching vibration mode, while the second SERS band can be related to double C-C bond stretching (37–39). We hypothesize that the AuNP surface interacts with the Lipid A units (the hydrophobic part of the LPS) leading to a strong enhancement of these bands. However, a numerical calculation should be performed to confirm this hypothesis (33–35). A detailed peak assignment is reported in the **Supplementary Table 3**.

In order to evaluate the LPS signal amplification provided by AuNPs, we calculated the ratio between the SERS and the Raman signal intensities, normalized to power, integration time and concentrations of the target molecule. By considering the intensity of the band at 1610 cm^-1^, we find that SERS provides gains of about 4 orders of magnitude compared to Raman.

Therefore, the AuNPs allows direct sensing of LPS, providing a good enhancement and reproducibility of the signal and avoiding the use of Raman reporters as previously reported (21). At the same time, the proposed approach allows for non-invasive and non-destructive characterization of the local chemical structure of the molecules attached to the AuNPs, avoiding the use of functionalization procedures.

Chemical specificity is an important advantage of the proposed SERS assay because the Raman peaks allow for easier distinction of different molecules and also different LPS types. In order to verify the specificity of the method, SERS characterization of LPS from two different bacteria, *E. coli* serotype O55:B5 and *Klebsiella pneumoniae*, has been performed (**Figures 5B, C**). Both Lipid A and O-antigen largely vary between the different LPS, representing the bacteria fingerprint (37–41). SERS analysis of the two LPS allows us to identify these differences and to distinguish between the two LPS types. Indeed, by comparing the two acquired SERS spectra, differences in peaks at 960 cm^-1^, 1025 cm^-1^ and 1610 cm^-1^ can be observed (**Figures 5B, C**). Since these signals are produced by the LPS lipidic domain, the observed differences can be associated with the different composition of the Lipid A in the two bacterial species (42, 43). Moreover, differences in signals at 1075 cm^-1^ and 1525 cm^-1^ are also present (**Figures 5B, C**). Since these signals are associated with the O-antigen, the differences in these Raman bands can be connected to the variability of the O-antigen among the bacterial species (44, 45).

### SERS Measurements of LPS-AuNPs and Limit of Detection

A valuable LPS sensor must be capable of detecting pure LPS in a board concentration range of with high chemical specificity, reproducibility and detection limit.

**Figure 6:**
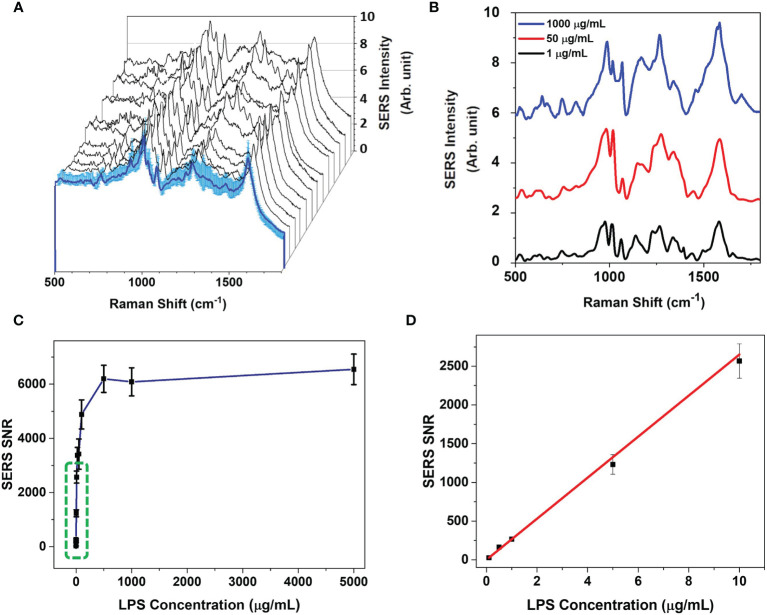
SERS measurements of the LPS-AuNPs. **(A)** Variability of raw SERS signals acquired on AuNP clusters related to a single concentration of LPS (1 mg/mL). **(B)** Average background-corrected SERS signals of AuNPs incubated with different LPS concentrations. LPS concentration (μg/mL) associated to each average SERS spectra are indicated on the right. Every SERS signal is obtained by averaging 30 spectra. **(C)** 1610 cm^-1^ peak SERS intensity signal to noise ratio (SNR) as a function of LPS concentration. The blue line represents the theoretical trend for concentration-dependent SERS signals. Green dashed square highlights the linear region of the trend, reported in the panel **(D)**. **(D)** Linear region of SNR on which LOD was estimated. Means ± SD of 1610 cm^-1^ peak SNR are reported. The red line represents the linear fit of the experimental data.

To evaluate sensitivity of our system, we performed SERS measurements of AuNPs (2 μL at a concentration of 1 mg/mL) incubated with 1 μL of LPS at different concentrations. At each concentration, the SERS signals were collected from 400 randomly selected regions of the substrate, and the mean spectrum was evaluated. **Figure 6A** shows 10 randomly selected spectra and the mean SERS spectrum for the LPS at 1 mg/mL. The spectra variability for this set of measurements was about 10%, which confirms that an excellent reproducibility of SERS technique. The high reproducibility associated to SERS signals could be due to the non-significant conformational changes that the molecules experience as they interact with the gold surface. According to our hypothesis, LPS molecules interact with AuNPs through their hydrophobic part (Lipid A) while the hydrophilic portion (O-antigen) is oriented towards the medium. This hypothesis is further strengthened by the presence of SERS bands at 973 cm^-1^ and 1610 cm^-1^, ascribed to the Lipid A, in all LPS concentration-dependent SERS spectra (**Figure 6B**). Furthermore, according to this assumption, LPS multilayer formation is not possible because, once the AuNP surface is saturated by LPS molecules, LPS phosphate groups in solution cannot interact with nanoparticles. On the other hand, citrate anions in the sample solution help LPS monomer formation and the consequent interaction with AuNPs. As a matter of fact, citrate anions are able to remove divalent cations in LPS micelles thereby weakening aggregate structures (46).

The SNR at each LPS concentration was evaluated by taking into account the ratio of the average of the peak intensity value at 1610 cm^-1^ to the SD of the spectral region between 1700 and 1800 cm^-1^. **Figure 6C** shows the measured SNR as a function of LPS concentration. The SERS spectra are reproducible at all the LPS concentrations. The signal intensities at lower LPS concentrations are good enough to detect and distinguish LPS at different concentrations from 0.1 to 1000 μg/mL, overcoming the limitation of the DLS measurements. It can be noted that the SNR values follow a positive increasing correlation with the LPS concentration in the range (0.1-100 μg/mL) and then reaches a plateau at LPS concentrations > 500 μg/mL. These results confirm the DLS data, suggesting that for concentrations > 500 μg/mL LPS uniformly covers the NP surface without the creation of multilayers.

The limit of detection (LOD), that is the concentration value at which the SNR becomes equal to unity (26), was estimated from a linear fit of the experimental data in the range (0.1-10 μg/mL) to be 2.6 ± 0.1 ng/mL (**Figure 6D**), comparable to the range obtained with colorimetric LAL assays (13). This LOD value has been obtained by considering the nominal concentration of LPS and AuNPs in the whole solution. However, the SERS system detects the signal from the few NPs available in the laser area - that is about 1000 AuNPs – providing a real LOD of 160 (± 10) fg/mL, that is lower than the detection limit of LAL assays (13). Furthermore, the proposed SERS approach does not suffer the limit of the fluorophore quenching, which is a serious issue in colorimetric and fluorogenic LPS detection assays, especially for nanoparticles, and allows to measure the number of LPS molecules directly on the single AuNP, without the use of SERS tag or functionalization procedures, as previously reported (21). Indeed, since the number of LPS molecules adsorbed on the NPs, in saturation condition, can be evaluated by the DLS measurements, as reported in the **Supplementary Table 1**, it is possible to estimate the SERS SNR as a function of the number of LPS molecules per AuNP (47). Therefore, we demonstrated that the minimum detectable number of LPS molecules per each AuNP is 5 ± 1, corresponding to about 0.16 ± 0.01 ag of LPS (see **Supplementary Table 2** and **Supplementary Figures 3**, **4**).

## Conclusions

In this study, an ultra-sensitive direct SERS sensing method was successfully developed, demonstrating the excellent ability of SERS technique to detect LPS directly on AuNPs without the use of any tag or functionalization procedure. We exploited the nanoparticles itself to detect the SERS signal of the endotoxin, to specifically identify the type of endotoxin and to quantitatively measure the amount of LPS on a single AuNP. Compared with existing approaches, SERS sensing showed good chemical specificity (allowing for an easy distinction of different molecules and different LPS types, without the use of functionalization procedures), broad detection range (0.1-1000 μg/mL), low detection limit (LOD of 5 LPS molecules per AuNP), high reproducibility and little sample volume required. The simple spectroscopic detection mechanism of SERS is low cost and reliable, and there is no need to select appropriate NP concentrations or functionalize/stain the sample before preforming measurements. Moreover, high enhancement of the Raman signal is inherently built into the assay, allowing for easy, direct and rapid detection of low LPS concentrations compared to other assays. The proposed sensor can be used to quantify the amount of LPS molecules attached on a single AuNP, that is 5 ± 1 LPS molecules at the minimum detectable signal, corresponding to about 0.16 ± 0.01 ag of LPS. These results pave the way to develop a robust bio-sensor based on the use AuNPs and SERS for LPS detection and quantification, with high sensitivity and specificity, which can complement the current LPS detection assays based on biological activity (recombinant Factor C assay and MAT).

## Data Availability Statement

The raw data supporting the conclusions of this article will be made available by the authors, without undue reservation.

## Author Contributions

AV and MM are joint first authors. ACDL, PI and DB conceptualized and designed the study. AV, MM, and SM designed, performed the experiments and analyses under the supervision of ACDL and PI. CT and IR performed DLS experiments. All the authors analyzed and discussed the data. AV and MM wrote the first draft. All authors contributed to the article and approved the submitted version.

## Funding

This work was supported by the EU Commission H2020 project ENDONANO (GA 812661) and PANDORA (GA671881). ACLL acknowledges financial support from the Italian Association for Cancer Research (AIRC) IG grant no. 21420, Italian Ministry for University and Research (project PON-NeON) and POR Campania FESR 2014/2020 (project POR-PLATT).

## Conflict of Interest

The authors declare that the research was conducted in the absence of any commercial or financial relationships that could be construed as a potential conflict of interest.

## Publisher’s Note

All claims expressed in this article are solely those of the authors and do not necessarily represent those of their affiliated organizations, or those of the publisher, the editors and the reviewers. Any product that may be evaluated in this article, or claim that may be made by its manufacturer, is not guaranteed or endorsed by the publisher.
