# Supplementary material for: SERS Sensing of Bacterial Endotoxin on Gold Nanoparticles
Source: Front Immunol. 2021 Oct 7;12:758410. doi: 10.3389/fimmu.2021.758410 (PMC8530015; doi:10.3389/fimmu.2021.758410)
Supplement: Supplementary file 1 [file DataSheet_1.pdf]

Supplementary Material

1 Supplementary Figures and tables

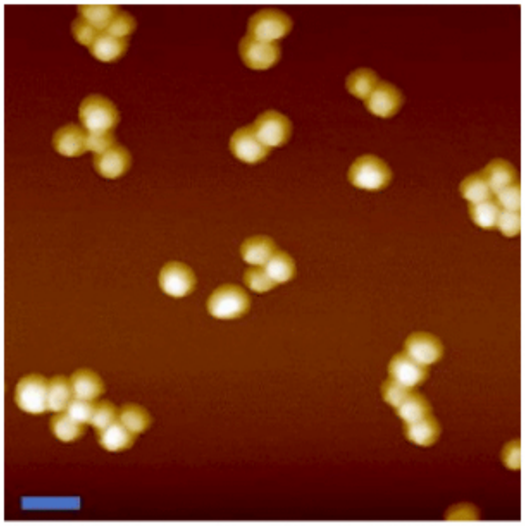

Supplementary Figure 1. AFM image of 50 nm AuNPs.

The AFM image shows the size and distribution of the AuNPs. Scale bar 100 nm.

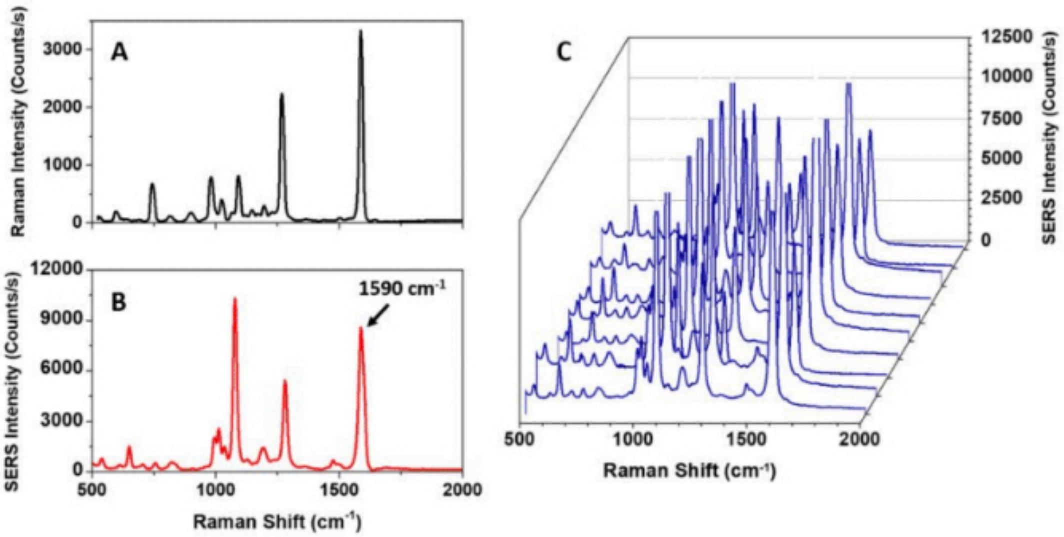

Supplementary Figure 2. Evaluation of the EF for the AuNPs

For EF calculation, 100 spectra were recorded from different locations of the substrate to create a statistically significant relevant data distribution. **(A)** Reference Raman spectrum acquired from the

BPT powder. The Raman spectra were acquired with a diode laser with emitting wavelength of 785 nm, power at the sample of 8.8 mW. The pinhole was set at 500 µm, the holographic grating at 600 lines/mm, and the slit at 200 µm. Acquisition of the Raman spectrum was performed with an integration time of 30 s. (B) Average SERS spectrum from the 50 nm AuNPs incubated with 1 mM BPT. SERS signals were collected over multiple points with an integration time of 1 s and a laser power of 0.1 mW. A total of 100 spectra were averaged. **(C)** Variability of raw BPT SERS signals acquired on 50 nm AuNPs.

Supplementary Table 1: Evaluation of the LPS corona around 50 nm AuNPs

LPS concentration (mg/mL)	Hydrodynamic diameter (nm)	s (nm)	LPS corona volume (nm^3)	s (nm^3)	LPS corona mass (ng)	s (ng)	LPS molecules	s
10	76	6	$2.3*10^5$	$0.5*10^5$	$9.6*10^{-8}$	$1.0*10^{-7}$	3000	2000
25	77	4	$2.4*10^5$	$0.4*10^5$	$1.1*10^{-7}$	$0.8*10^{-7}$	3000	2000
50	81	5	$2.8*10^5$	$0.5*10^5$	$1.7*10^{-7}$	$1.0*10^{-7}$	5000	3000
100	85	3	$3.2*10^5$	$0.4*10^5$	$2.3*10^{-7}$	$0.8*10^{-7}$	7000	2000
500	89	6	$3.7*10^5$	$0.7*10^5$	$3.0*10^{-7}$	$1.2*10^{-7}$	9000	3000
1000	88	4	$3.6*10^5$	$0.5*10^5$	$2.8*10^{-7}$	$0.9*10^{-7}$	9000	3000
5000	87	5	$3.5*10^5$	$0.6*10^5$	$2.7*10^{-7}$	$1.0*10^{-7}$	8000	3000

In order to estimate the number of LPS molecules composing the corona around a single 50 nm AuNP ($\#LPS_{AuNP}$), the total amount of LPS attached to 2 µL of 50 nm AuNPs at 1 mg/mL (corresponding to ~ 10^9 NPs) and LPS molecules per single AuNP were calculated (21) by: $TOT\ LPS\ mass = m_{LPS\ corona} * \#NPs\ (10^9\ NPs)$; $\#LPS_{AuNP} = m_{LPS\ corona}/M_{LPS}$; M_{LPS} = LPS molecular weight (20 kDa ~ $3.32*10^{-20}$ g). The green box indicates the LPS concentrations at which the AuNP surface

Supplementary Table 2. LPS molecules in the spot area, LPS molecules per single AuNP and LPS amount per single AuNP as estimated by the evaluation of the SERS SNR.

LPS concentration (mg/mL)	LPS molecules in the laser spot area	s	LPS molecules per AuNP	s	LPS amount per AuNP (ag)	s (ag)
0.1	$3.8*10^4$	$0.9*10^4$	40	10	1.2	0.3
0.5	$2.3*10^5$	$0.6*10^5$	230	60	8.6	1.9
1	$3.8*10^5$	$0.9*10^5$	370	90	12.3	3.1
5	$1.7*10^6$	$0.4*10^6$	1700	400	56.2	14.3
10	$3.6*10^6$	$0.9*10^6$	3500	900	118.8	29.9
25	$4.8*10^6$	$1.2*10^6$	5000	1000	155.4	39.5
50	$4.9*10^6$	$1.2*10^6$	5000	1000	158.6	40.1
100	$6.9*10^6$	$1.8*10^6$	7000	2000	225.1	57.1
500/1000/5000	$8.9*10^6$	$2.3*10^6$	9000	2000	289.3	73.5

In order to estimate the number of LPS molecules on AuNPs inside the laser spot area ($\#LPS_{laser\ area}$) for each LPS concentration, we first evaluated the number of AuNPs in the laser area as follows: $\#AuNPs_{laser\ \ area} = \frac{A_{laser}}{A_{50nm\ AuNP}} = \frac{\pi w^2}{\pi R^2}$
where $\pi w^2$is the laser spot area, w is the beam waist measured to be about 800 nm, and πR^2 is the AuNP area. The total number of LPS molecules hit by the laser beam was then calculated by:

$$\#LPS_{laser\ area\_sat} = \#AuNPs_{laser\ area} \cdot \#LPS_{AuNP\_sat}$$

where $\#LPS_{AuNP\_sat}$ is the estimated number of LPS molecules on a single AuNP in saturation conditions (as determined by DLS measurements). The estimated values are reported in the Table.
Subsequently, the SERS signal to noise ratio (SNR_{sat}) corresponding to $\#LPS_{laser\ area\_sat}$ at the saturation condition was measured by averaging the SNR related to LPS concentrations of 500, 1000 and 5000 μg/mL (where the LPS corona is uniform around the AuNPs).
The estimation of $\#LPS_{laser\ area}$ as a function of the LPS concentration was obtained by:

$$SNR_{sat}/\#LPS_{laser\ area\_sat} = SNR_{[LPS]}/\#LPS_{laser\ area}$$

where $SNR_{[LPS]}$ is the SERS signal to noise ratio measured at different LPS concentrations. By dividing $\#LPS_{laser\ area}$ by $\#AuNPs_{laser\ \ area}$, the number of LPS molecules per AuNP was estimated for each concentration. The Langmuir adsorption isotherm fit used to associate LPS molecules per single AuNP to the corresponding LPS concentration is depicted in the Supplementary Figure 3 (41).

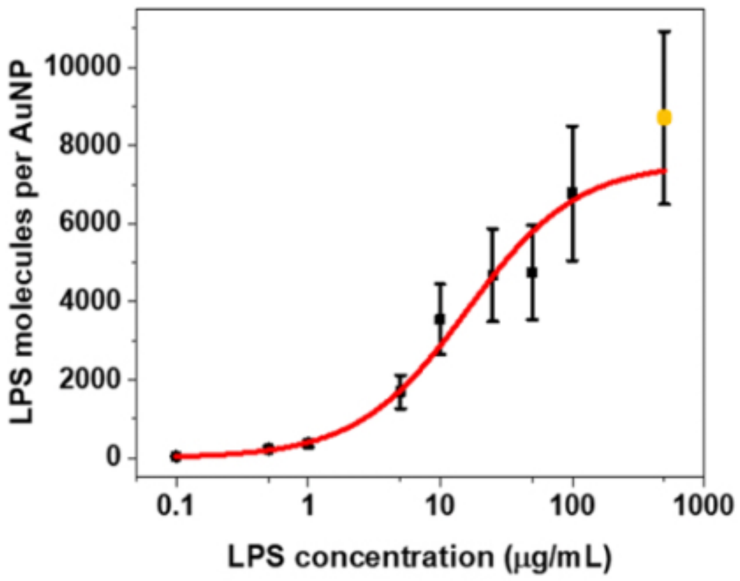

Supplementary Figure 3. Number of LPS molecules per single AuNP.

The number of LPS molecules amount per single AuNP are expressed as a function of LPS concentration (log scale). The orange point represents the average number of LPS molecules per single AuNP corresponding to the saturation condition. The red line represents the Langmuir adsorption isotherm fit.

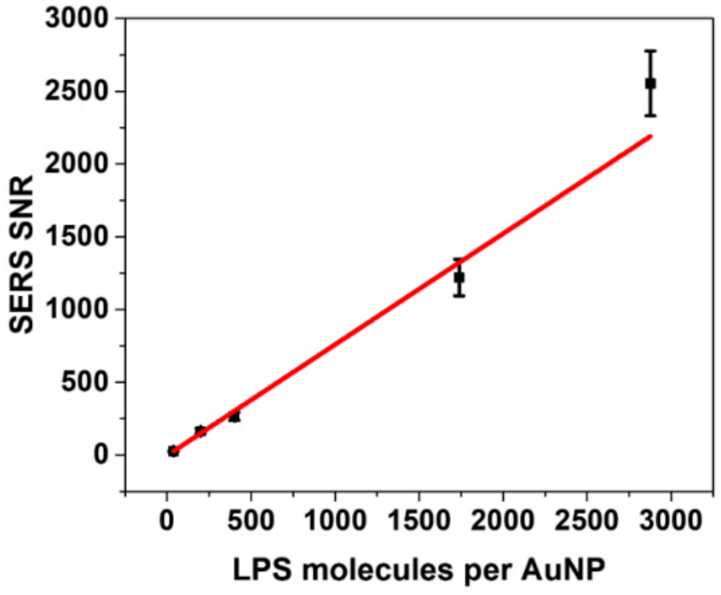

Supplementary Figure 4. The SERS SNR as a function of the LPS molecules per single AuNP.

Based on the calculations reported in the Supplementary Tables 1 and 2, it is possible to express the SERS SNR as a function of the number of LPS molecules per single AuNP. From a liner fit of the

data in the linear region (LPS concentration range of 0.1-10 μg/mL, red line), the LOD of our system was estimated to be 5 LPS molecules per single AuNP.

Supplementary Table 3. Raman and SERS peaks assignment of LPS

Bands (cm^{-1})	Vibrational Mode	LPS Component
535	δC–O–C (glycosidic linkage), Bending CH_2	O-Antigen, Core
740	Symmetric $\nu(CH_3)_3$	Lipid A
960	Bending CH νC–O–C	Lipid A
973	Bending CH νC–O–C	Lipid A
1025	Bending CH	Lipid A
1065 – 1075	νC-C, νC-O	O-Antigen, Core
1115	νC–C	Lipid A
1177	δC–O–C (glycosidic linkage)	Lipid A
1256	δ(=CH), Bending CH	Lipid A, O-Antigen, Core
1300 - 1320	δC-H, Bending CH_2	O-Antigen, Core
1350 - 1370	Bending CH	O-Antigen, Core
1440 - 1470	Scissoring CH_2/CH_3 δC–O–C (glycosidic linkage), δC–C-C, δC–O–H	Lipid A O-Antigen, Core
1525	νC=O	Lipid A
1610 - 1640	νC=C	Lipid A
1726	νC=O	Lipid A

δ – deformation; ν – stretching
